# Prediction of individual COVID-19 diagnosis using baseline demographics and lab data

**DOI:** 10.1038/s41598-021-93126-7

**Published:** 2021-07-06

**Authors:** Jimmy Zhang, Tomi Jun, Jordi Frank, Sharon Nirenberg, Patricia Kovatch, Kuan-lin Huang

**Affiliations:** 1 Department of Genetics and Genomic Sciences, Center for Transformative Disease Modeling, Tisch Cancer Institute, Icahn Institute for Data Science and Genomic Technology, Icahn School of Medicine At Mount Sinai, New York, NY 10029 USA; 2 Queens High School for the Sciences At York College, Jamaica, NY 11451 USA; 3 Department of Hematology and Medical Oncology, Icahn School of Medicine At Mount Sinai, New York, NY 10029 USA; 4 Outco Inc, San Francisco, CA 94104 USA; 5 Scientific Computing, Icahn School of Medicine At Mount Sinai, New York, USA

**Keywords:** Infectious diseases, Machine learning, Programming language

## Abstract

The global surge in COVID-19 cases underscores the need for fast, scalable, and reliable testing. Current COVID-19 diagnostic tests are limited by turnaround time, limited availability, or occasional false findings. Here, we developed a machine learning-based framework for predicting individual COVID-19 positive diagnosis relying only on readily-available baseline data, including patient demographics, comorbidities, and common lab values. Leveraging a cohort of 31,739 adults within an academic health system, we trained and tested multiple types of machine learning models, achieving an area under the curve of 0.75. Feature importance analyses highlighted serum calcium levels, temperature, age, lymphocyte count, smoking, hemoglobin levels, aspartate aminotransferase levels, and oxygen saturation as key predictors. Additionally, we developed a single decision tree model that provided an operable method for stratifying sub-populations. Overall, this study provides a proof-of-concept that COVID-19 diagnosis prediction models can be developed using only baseline data. The resulting prediction can complement existing tests to enhance screening and pandemic containment workflows.

## Introduction

As of late January, 2021, nearly 100 million cases of COVID-19 have been confirmed globally, with over two million deaths^1^, placing tremendous strain on healthcare systems around the world. The COVID-19 pandemic has necessitated mass testing as a starting point for tracking and ultimately containing the spread of the disease^2–4^. Fast, widespread testing would help to identify infected individuals early on and slow disease transmission through early public health interventions, such as quarantine and contact tracing^5^.

However, current COVID-19 diagnostic tests are imperfect. RT-PCR based assays are widely regarded as the gold standard^6,7^ but could occasionally yield false findings and the return of results often take multiple days^7–9^. Further, recent evaluations by the UK National COVID-19 Scientific Advisory Panel revealed that the sensitivity of ELISA and lateral flow immunoassay (LFIA) devices were 85% and less than 70% compared to RT-PCR, depending on the assayed time points^10^. Based on the Bayes’ theorem, medical tests that are not perfectly sensitive and specific can yield undesired ratios of true to false findings, especially when widespread testing of the general population is performed^11^. In such circumstances, combining tests with other prioritization metrics can improve accuracy and help allocate testing for high-risk individuals.

Multiple pandemic scenarios need to be addressed with enhanced capacity to quickly or repeatedly screen large populations and deploy containment strategies, including (1) high-exposure-risk populations needing repeated testing and monitoring^12^, (2) under-resourced regions lacking personnel, testing kits, and biosafety facilities^13,14^, and (3) the monitoring hotspots for local outbreaks^2,15^. Predictive models for the risk of testing COVID positive could facilitate testing resource allocation and contact tracing procedures.

Until other measures to counteract COVID-19 become widely available (ex. effective and mass-distributed vaccines)^16,17^, effective screening methods are pivotal in containing the pandemic. Establishing a machine learning-based tool that relies only on baseline data will help in prioritizing sub-populations for COVID-19 testing, and is urgently needed to relieve the burden of large-scale screening when testing capacity may be limited.

## Methods

### Study setting and data sources

The study was conducted based on patient cohorts from the Mount Sinai Health System, which comprises 8 hospitals and more than 410 ambulatory practice locations in the New York metropolitan area. Data were derived from clinical records from Mount Sinai facilities using the Epic electronic health record (Epic Systems, Verona, WI). Data were directly extracted from Epic’s Clarity and Caboodle servers.

The curation of EHR data is as previously described^18,19^. Briefly, in the setting of the COVID-19 pandemic, the Mount Sinai Data Warehouse (MSDW) developed and released a de-identified data set encompassing all COVID-19 related patient encounters within the Mount Sinai system, accompanied by selected demographics, comorbidities, vital signs, medications, and lab values. De-identification was performed in accordance with the Safe Harbor subsection of the HIPAA Privacy Rule^20^. The MSDW dataset captured any patient encounters at a Mount Sinai facility with any of the following: a COVID-19 related encounter diagnosis, a COVID-19 related visit type, a SARS-CoV-2 lab order, a SARS-CoV-2 lab result, or a SARS-CoV-2 lab test result from the New York State Department of Health’s Wadsworth laboratory. As part of de-identification, all patients over the age of 89 had their age set to 90. Initial vital signs were the first vital signs documented for the encounter. We defined initial labs as the first lab value within 24 h of the start of the encounter.

For this study, we included all adult patients (18 or older) who had a COVID-19 test up to June 2nd, 2020. We split the cohort into a training set (encounters up to April 13th, 2020) and a test set (encounters from April 14th to June 2nd, 2020).

This study utilized de-identified data extracted from the electronic health. The Institutional Review Board (IRB) of the Mount Sinai School of Medicine (MSSM), in accordance with Mount Sinai’s Federal Wide Assurances (FWA#00005656, FWA#00005651) to the Department of Health and Human Services approved this human subject research. The IRB has determined that this research involves no greater than minimal risk and approved the waiver for informed consent. The MSSM IRB approved the request for Waiver of Authorization for use and disclosure of PHI for this project under expedited review procedure category 5. All analyses were carried out in accordance with relevant guidelines and regulations.

### Machine learning models

We implemented a machine learning framework to build a COVID-19 diagnosis predictor using three different classification algorithms: logistic regression, random forest, and eXtreme Gradient Boosting (XGBoost). The goal of this predictor was to predict whether or not a patient would test positive for SARS-CoV-2 from an RT-PCR test. Tests reported as “presumptive positive” were classified as positive results for the purposes of training and validation.

### Feature selection

A total of 38 features were used in the final models. Since the models would be applied as screening tools in populations not receiving specialized medical attention, we chose features that would be readily available or easily obtained such as patient demographics, comorbidities, and common lab values. Clinical features were selected by a clinician involved in the care of COVID-19 patients (TJ). In order to maximize feature inclusion while removing any features unlikely to be predictive, we omitted any features containing over 70% missing values. The values of categorical features were label encoded and converted into numerical form (0, 1, 2, 3, …). Ordinal categorical features, e.g. smoking, were encoded based on the sequential relation between discrete values, e.g. ‘NEVER’ < ‘FORMER’ < ‘CURRENT’. Nominal categorical features, e.g. race, were encoded based on the order in which discrete values appeared in the data.

### Model training, cross-validation, and hyperparameter optimization

We stratified the patient population in the MSDW dataset into a training set to train the prediction models and a test set to test each model’s performance. We used the patients tested through April 13, 2020 (N = 12,476) as the training set and the patients tested from April 13, 2020 through June 2, 2020 (N = 19,263) as the test set.

Since random forest models and logistic regression models are incompatible with missing values, we created a separate training set and test set without missing values. Missing values for each feature were imputed by the median value (for continuous variables) or the most frequently occurring value (for categorical variables) for that feature in its respective dataset.

We trained the XGBoost model on the unimputed training set, and we trained the random forest and logistic regression models on the imputed training set. We applied a randomized search with sixty rounds of five-fold cross validation to identify the optimal hyperparameters for each of the three models. For the XGBoost model, we optimized ‘n_estimators’, ‘learning_rate’, ‘subsample’, ‘max_depth’, ‘colsample_bytree’, ‘min_child_weight’, and ‘gamma.’ For the random forest model, we optimized ‘max_features’, ‘min_samples_split’, ‘min_samples_leaf’, ‘n_estimators’, ‘max_depth’, and ‘bootstrap.’ For the logistic regression model, we optimized ‘C’, ‘penalty’, ‘solver’, and ‘max_iter.’ The full list of final hyperparameters for these three models is available in Supplementary Table S1A–C.

To determine whether changing the number of folds (k) during the randomized search cross validation would affect predictive performance, we retrained the same four models using ten-fold cross validation instead of five-fold cross validation. However, due to similar predictive performance and hyperparameter selection between the five-fold and ten-fold cross validated models, we conducted our final analyses on the five-fold cross validated models. The performance of the ten-fold cross validated models is shown in the AUC-ROC curves available in Supplementary Fig. S1.

### Model testing

We then evaluated the performance of each model on their respective test sets. We tested the XGBoost model on the unimputed test set, and we tested the random forest and logistic regression models on the imputed test set. Each model derived and utilized class probabilities to predict COVID-19 diagnosis with a threshold of ≥ 0.5, in which predicted probabilities greater than the threshold corresponded to predicted positive diagnosis.

The performance of each model was evaluated in terms of classification accuracy, negative predictive value, precision, recall/sensitivity, and area under the receiver operating characteristic curve (AUC-ROC). Feature importance within each model was determined using SHAP (SHapley Additive exPlanations)^21^. SHAP is a unified, model-agnostic, and game-theoretic approach of computing Shapley values to explain the contribution of each feature to a given prediction. SHAP values are calculated by using additive feature attribution methods to approximate the Shapley values of the model’s conditional expectation function^21^. We used the LinearExplainer to calculate the SHAP values of the logistic regression model, and we used the TreeExplainer to calculate the SHAP values of the XGBoost and random forest models.

### Development of an interpretable, single-tree XGBoost model

XGBoost is widely regarded as a high-performance algorithm due to its reliance on gradient tree boosting, an ensemble technique that enables XGBoost to add new trees to correct errors made by existing trees^22^. However, since XGBoost combines results across multiple trees when making predictions, the decision algorithm utilized by XGBoost models is often difficult to interpret.

Therefore, we also developed a more interpretable XGBoost model to predict COVID-19 diagnosis by limiting the number of trees to 1, which reduces the complexity of the model to yield a single decision tree that can be visualized as a simple binary flowchart. Although a single-tree XGBoost model can suffer slightly in predictive power as compared to a multi-tree model, a single decision tree can reveal a much simpler and more clinically applicable decision algorithm^23^.

This single-tree XGBoost model was trained on the unimputed training set and five-fold cross validated over sixty rounds in a randomized search to determine optimal hyperparameters. The hyperparameter search space used during the randomized search for the single-tree XGBoost model was the same as that of the multi-tree XGBoost model, with the exception of the n_estimators and max_depth parameters, which were manually set to 1 and 4, respectively. The full list of hyperparameters for the single-tree XGBoost model is available in Supplementary Table S1D. Subsequently, the single-tree XGBoost model was tested on the unimputed test set and evaluated alongside the other three models.

## Results

### Predictive models of COVID-19 diagnosis

The full MSHS study cohort used to train and test our models contained a total of 31,739 adult patients who had a COVID-19 test up to June 2nd, 2020 (Supplementary Fig. S2 and Table S2). To demonstrate the utility of training a model based on past data that have prospective predictive values, this cohort was split based on encounter dates into a training set (N = 12,476, patients tested through April 13, 2020; of whom 6884 tested positive and 5592 tested negative) and a test set (N = 19,263, patients tested from April 13, 2020 through June 2, 2020; of whom 2940 tested positive and 16,323 tested negative).

**Figure 1:**
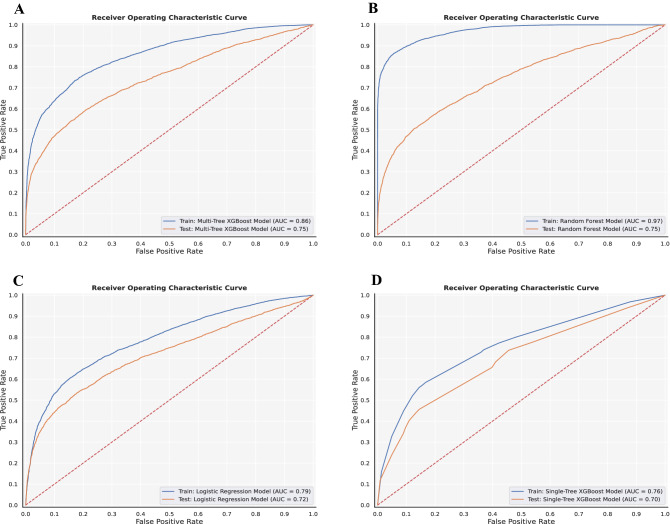
Receiver operating characteristic curves. Receiver operating characteristic curves of (**A**) the multi-tree XGBoost model, (**B**) the random forest model, (**C**) the logistic regression model, and (**D**) the single-tree XGBoost model in their respective train and test sets.

We conducted predictive modeling of COVID-19 diagnosis using the described data. Five-fold cross-validated XGBoost, random forest, and logistic regression models predicting COVID-19 diagnosis were trained on the training set and tested on the test set (Methods). We evaluated and compared the performance of the multi-tree XGBoost model, the random forest model, and the logistic regression model in correctly predicting COVID-19 diagnosis of patients within their respective test sets: the XGBoost model was tested on the unimputed test set while the random forest and logistic regression models were tested on the imputed test set (Methods). The predictive performance of each of the three models is provided in Table 1, and the AUC-ROC curves of the prediction models are shown in Fig. 1A–C. The multi-tree XGBoost model (AUC score = 0.75) and the random forest model (AUC score = 0.75) achieved similar performance, while the logistic regression model performed slightly worse (AUC score = 0.73).

**Table 1:** Predictive performance of each COVID-19 diagnosis prediction model in the test set.

Model	Accuracy (%)	Precision (%)	Sensitivity (%)	Specificity (%)	Negative predictive value (%)	AUC-ROC (%)
Multi-tree XGBoost classifier	77.66	35.54	57.04	81.37	91.32	74.67
Random forest classifier	79.10	37.21	53.71	83.67	90.94	74.53
Logistic regression classifier	79.05	36.66	51.19	84.07	90.53	71.75
Single-tree XGBoost classifier	79.37	36.11	45.68	85.44	89.73	69.64

### Feature importance using SHAP

**Figure 2:**
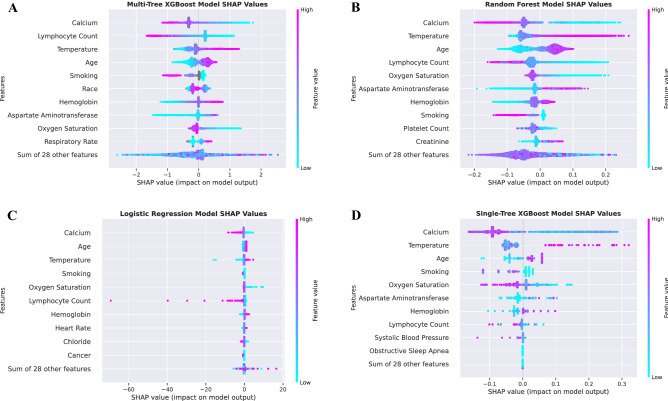
Feature importance across all four models. SHapley Additive exPlanations (SHAP) beeswarm plots of (**A**) the multi-tree XGBoost model, (**B**) the random forest model, (**C**) the logistic regression model, and (**D**) the single-tree XGBoost model. Features are ranked in order of decreasing mean absolute SHAP value. The SHAP value distribution of the top ten most predictive features in each model is displayed. The SHAP values on the x-axis of the tree-based models have log-odds units, while the SHAP values on the x-axis of the logistic regression model have probability units. For each feature, each patient is represented by a single point, and the x-position of each point represents the impact of that feature on a given patient. The color of each point corresponds to the patient’s value for that feature, ranging from low to high.

To evaluate the key features predictive of COVID-19 diagnosis, we assessed the contribution of each input variable to the performance of each machine learning model as determined by SHAP (SHapley Additive exPlanations). The SHAP beeswarm plots for all four models are shown in Fig. 2, and features were ranked in order of decreasing average importance (mean absolute SHAP value). The SHAP values on the x-axis of the tree-based models have log-odds units, while the SHAP values on the x-axis of the logistic regression model have probability units. Notably, the features that had high SHAP values across multiple models were calcium, temperature, age, lymphocyte count, smoking, hemoglobin, aspartate aminotransferase, and oxygen saturation.

### Important features in the multi-tree XGBoost model

Since the multi-tree XGBoost model tied for the highest AUC score in the test set and outperformed the random forest model during cross-validation, we also assessed the feature importance of the multi-tree XGBoost model using XGBoost’s built-in feature importance metrics. Features were ranked according to their relative contribution, also known as gain: the average training loss reduction gained by using a particular feature to split the data, as calculated by the log loss function^24^. The relative gain of a feature (the gain provided by that feature divided by total gain across all features) demonstrates the relative importance of that feature compared to other features in the model. The relative gain of each feature in the multi-tree XGBoost model is summarized in Supplementary Fig. S3.

### Identifying an optimal probability threshold for patient screening

**Figure 3:**
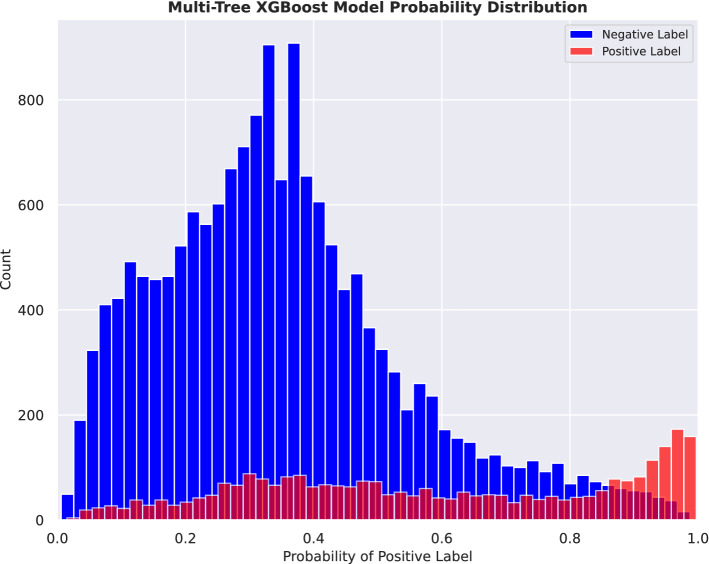
Distribution of predicted class probabilities derived from the multi-tree XGBoost model. Blue bars represent patients who tested RT-PCR negative, and red bars represent patients who tested RT-PCR positive.

In the multi-tree XGBoost model, the predicted class probabilities of COVID-19 diagnosis across all patients in the test set revealed a distribution close to actual outcome for patients that tested negative (median = 0.33, SD = 0.19), while the distribution of patients that tested positive showed a wider range of probabilities (median = 0.58, SD = 0.28), as shown in Fig. 3. Such a probability score can prioritize individuals for testing. For example, above the probability threshold of 0.85, we identified 854 patients that tested positive and 331 patients that tested negative in the test set. The positive-test rate of 72% for this prioritized sub-population is notably higher than the 15% overall positive rate in the entire test set patient population.

### Single-tree XGBoost model performance

**Figure 4:**
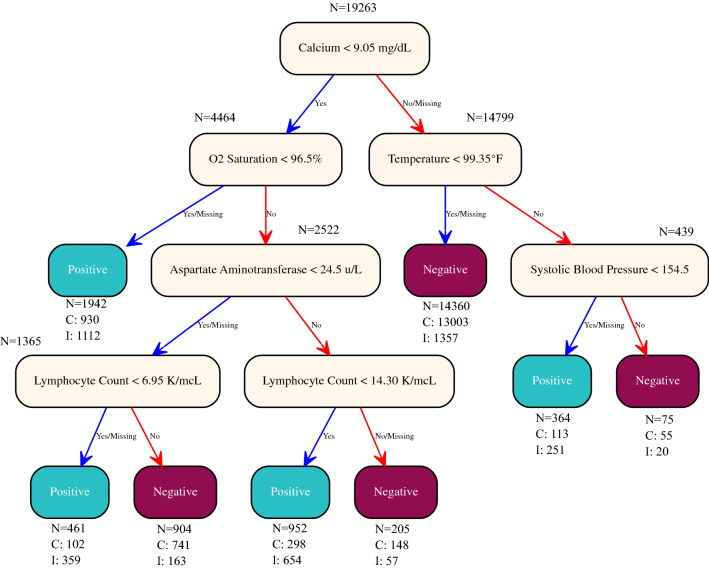
A clinically applicable decision algorithm used by the single-tree XGBoost model. N = number of patients in a class; C = number of patients correctly classified; I = number of patients incorrectly classified.

Compared to the multi-tree XGBoost model, the single-tree XGBoost model performed slightly worse with an AUC score of 0.69 in the test set. The performance of the single-tree XGBoost model in predicting COVID-19 diagnosis in the test set is also provided in Table 1, and its corresponding AUC-ROC curve is provided in Fig. 1D. Despite suffering slightly in predictive strength, the single-tree XGBoost model relies only on a single decision tree, which can be easily interpreted and adapted into a simple, clinically applicable decision rule, as shown in Fig. 4.

## Discussion

In this study, our machine learning framework provides a proof-of-concept for predicting COVID-19 diagnosis (RT-PCR test result) relying only on baseline demographics, comorbidities, vitals, and lab values. The predictive models could prioritize sub-populations for COVID-19 diagnosis in situations where testing capacity may be limited, or they could also be used in conjunction with clinical judgment or other predictive models (ex. based on mobile phone data) to verify RT-PCR test results. Our predictive models also identified key clinical features that correlate with a positive diagnosis, providing insights on efficient patient stratification and population screening. Moreover, the decision algorithm derived from the single-tree XGBoost model provides a simple, clinically operable method of stratifying sub-populations that can be replicated in other settings.

Each of our four models revealed several key clinical variables predictive of positive RT-PCR test result. One key finding was that serum calcium levels was the most predictive feature of COVID-19 diagnosis across all four models; concurrently, previous studies identified serum calcium as a biomarker of clinical severity and poor prognosis in COVID-19 patients^25,26^. Our single-tree XGBoost model even uses serum calcium level < 9.05 mg/dL as the first split in the decision tree (Fig. 4), which also correlates with previous findings confirming the prevalence of hypocalcemia in severe COVID-19 patients^25^. Notably, a serum calcium test typically has a rapid turnaround time within a day and thus may be valuable in complementing existing tests.

The development of acute respiratory distress syndrome (ARDS) and/or sepsis, along with their associated symptoms, have also been shown to be a key indicator of positive COVID-19 diagnosis^2,27^. While the datasets used to train and test the machine learning models did not directly include symptoms of COVID-19, the trained models prioritized features that may contribute to COVID-19 positivity in both symptomatic and asymptomatic individuals. Our four models identified features such as age, lab values (calcium levels, aspartate aminotransferase levels), comorbidities (smoking), vitals (oxygen saturation, temperature), and hematologic features (lymphocyte count, hemoglobin levels) to be predictive of positive diagnosis. Many of these identified features have been previously reported as markers of COVID-19 severity. For instance, abnormal liver function tests, which includes elevated levels of AST, have previously been found to be a marker of poor clinical outcome in COVID-19 patients^26–28^. Decreased white blood cell count (lymphopenia) and hemoglobin levels (anemia) have been confirmed to be correlated with low serum calcium levels (hypocalcemia) and severe COVID-19 disease progression^6,25,26,29^.

We highlight multiple conscious design choices in constructing the machine-learning models for practical reasons. First, a split of train vs. test sets based on date mimics real-world situations, in which predictive models can only be trained on past data to facilitate prospective predictions. The samplings for tested individuals likely differ across the two time periods in New York City, and the ability of our model to predict a prospective cohort based on past data provide confidence to this approach. Second, our models rely solely on baseline features that can be easily obtained at initial patient encounter, which will have significant practical implications in prioritizing sub-populations for testing in areas with limited test kits or testing capacity. Indeed, the resulting models remain a proof-of-principal; given the different sampling populations and available lab tests, best performance of COVID-19 diagnosis prediction can likely be achieved if each testing site derive its own predictive model. Our codes are made openly available for future implementations.

Our machine learning models had several limitations. First, the variable predicted by the models was RT-PCR test result, which, despite being widely regarded as the gold standard^6,7^ for the diagnosis of COVID-19, is still prone to error and limits the peak performance of the model. We note that if data from a new test outperforming the RT-PCR test become available, our predictive models can be adapted to leverage the results of the new test. Second, the data contained high proportion of missing values in certain variables, especially in lab tests, which may have contributed to the low precision in our models. Although XGBoost models are compatible with missing values, the inclusion of more complete patient records may improve the performance of subsequent model versions. Finally, as more data becomes available, our machine learning models could be retrained and validated in other settings (e.g., health systems, testing sites, schools) to evaluate model performance and utility across populations.

Overall, this study provided a proof-of-concept that predictive models of COVID-19 diagnosis can be developed to help prioritize sub-populations for more efficient screening or complement existing tests. Given that the COVID-19 pandemic continues to affect large fractions of populations^2,15^, efficient screening of COVID-19 diagnosis, identification of high risk factors of COVID-19 positivity, and stratification of patient populations will play a crucial role in the allocation of limited testing resources for efficient testing and facilitation of patient management.

## Supplementary Information

Supplementary Information.

## Data Availability

The summarized data are available in Supplementary Tables. The implemented code is available at https://github.com/Huang-lab/covid19-diagnosis-prediction.
