# Supplementary material for: Prediction of individual COVID-19 diagnosis using baseline demographics and lab data
Source: Sci Rep. 2021 Jul 6;11:13913. doi: 10.1038/s41598-021-93126-7 (PMC8260732; doi:10.1038/s41598-021-93126-7)
Supplement: Supplementary file 1 — Supplementary Information. [file 41598_2021_93126_MOESM1_ESM.pdf]

Supplementary Information for "Prediction of individual COVID-19 diagnosis using baseline demographics and lab data"

Jimmy Zhang[1,2], Tomi Jun[3], Jordi Frank[4], Sharon Nirenberg[5], Patricia Kovatch[5], Kuan-lin Huang[1#]

[1] Department of Genetics and Genomic Sciences, Center for Transformative Disease Modeling, Tisch Cancer Institute, Icahn Institute for Data Science and Genomic Technology, Icahn School of Medicine at Mount Sinai, New York, NY 10029, United States.

[2] Queens High School for the Sciences at York College, Jamaica, NY 11451, United States.

[3] Department of Hematology and Medical Oncology, Icahn School of Medicine at Mount Sinai, New York, NY 10029, United States.

[4] Outco Inc., San Francisco, CA 94104, United States.

[5] Scientific Computing, Icahn School of Medicine at Mount Sinai

[#]Corresponding Author:

Kuan-lin Huang, Ph.D.
Department of Genetics and Genomic Sciences
Icahn School of Medicine at Mount Sinai
New York, NY 10029
Email: kuan-lin.huang@mssm.edu

Supplementary Table 1: List of final hyperparameters

A)

Hyperparameter	Value
Number of trees (n_estimators)	355
Maximum depth (max_depth)	3
Learning rate (learning_rate)	0·07
Percentage of samples used per tree (subsample)	0·494
Percentage of features used per tree (colsample_bytree)	0·895
Minimum sum of instance weight needed per child (min_child_weight)	19
Gamma regularization parameter (gamma)	0·1

B)

Hyperparameter	Value
Number of trees (n_estimators)	289
Maximum depth (max_depth)	30
Minimum number of samples required to split an internal node (min_samples_split)	10
Minimum number of samples required per leaf node (min_samples_leaf)	2
Number of features considered when looking for best split (max_features)	Auto
Bootstrap sampling (bootstrap)	True

C)

Hyperparameter	Value
Algorithm used (solver)	Liblinear
Penalization norm (penalty)	L2
Maximum number of iterations for solver to converge (max_iter)	2000
Inverse of regularization strength (C)	10000

D)

Hyperparameter	Value
Number of trees (n_estimators)	1
Maximum depth (max_depth)	4
Learning rate (learning_rate)	0·26
Percentage of samples used per tree (subsample)	0·595
Percentage of features used per tree (colsample_bytree)	0·936
Minimum sum of instance weight needed per child (min_child_weight)	17
Gamma regularization parameter (gamma)	1

Hyperparameters used in (A) the multi-tree XGBoost model, (B) the random forest model, (C) the logistic regression model, and (D) the single-tree XGBoost model. All hyperparameters were selected through a randomized search with five-fold cross validation, with the exception of the 'n_estimators' and 'max_depth' parameters in the single-tree XGBoost model, which were manually set so that a single-tree model could be produced (**Methods)**. All unlisted parameters were initialized to their algorithm's default values.

Supplementary Figure 1: Receiver operating characteristic curves of ten-fold cross validated models

A)

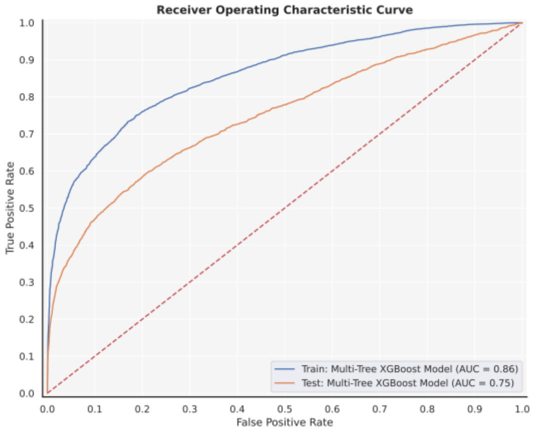

B)

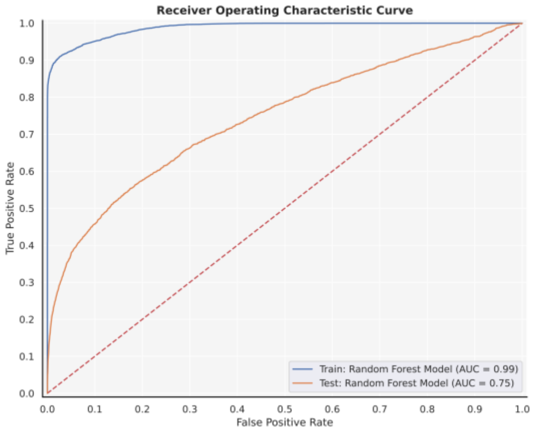

C)

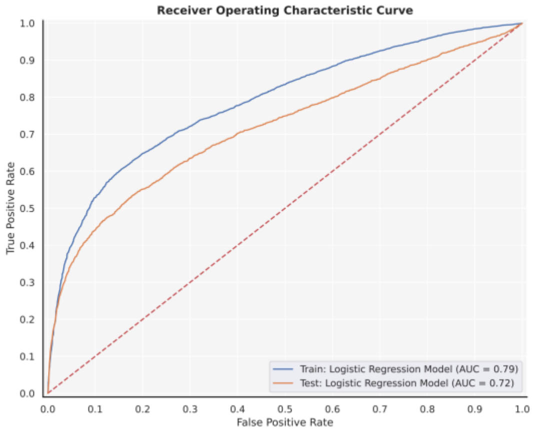

D)

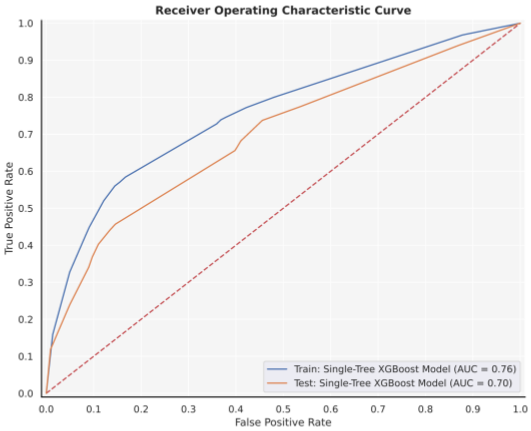

Receiver operating characteristic curves of (A) the multi-tree XGBoost model, (B) the random forest model, (C) the logistic regression model, and (D) the single-tree XGBoost model in their respective train and test sets (ten-fold cross validation used during hyperparameter optimization). Due to similar performance and similar hyperparameter selection across both the five-fold and ten-fold cross validated models, subsequent analyses were conducted using only the five-fold cross validated models.

Supplementary Figure 2: Flow diagram of included patients

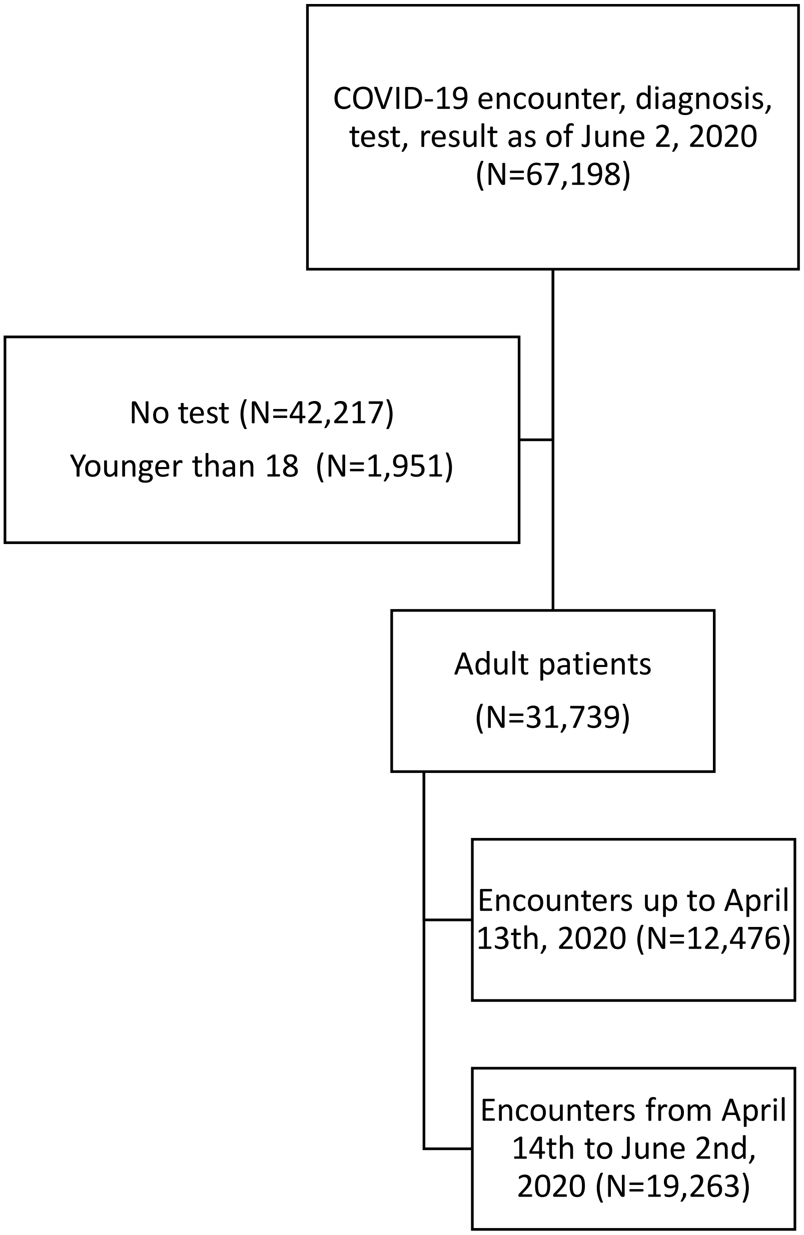

Supplementary Table 2: Descriptive cohort characteristics

Variable	All patients (N=31,739)
Age (yrs)	54 (36 - 68)
Male	15588 (49.1%)
Female	16134 (50.8%)
Asian	1941 (6.1%)
Hispanic	6426 (20.2%)
Non-Hispanic Black	6831 (21.5%)
Non-Hispanic White	10171 (32%)
Other race/ethnicity	4087 (12.9%)
Current smoker	2485 (7.8%)
Former smoker	5884 (18.5%)
Never smoker	15843 (49.9%)
Body mass index (kg/m2)	26.96 (23.49 - 31.38)
Hypertension	6988 (22%)
Diabetes	3947 (12.4%)
Coronary artery disease	2709 (8.5%)
Heart failure	1554 (4.9%)
Atrial fibrillation	1440 (4.5%)
Chronic kidney disease	1992 (6.3%)
Chronic obstructive pulmonary disorder	869 (2.7%)
Asthma	1632 (5.1%)
Obesity	1896 (6%)
Cancer	4042 (12.7%)
Chronic viral hepatitis	307 (1%)
Other liver disease	648 (2%)
Obstructive sleep apnea	511 (1.6%)
HIV	583 (1.8%)
Temperature (°F)	98.2 (97.7 - 98.8)
Heart rate (bpm)	89 (77 - 103)
Systolic blood pressure (mmHg)	131 (117 - 146.75)
Respiratory rate (bpm)	18 (18 - 20)
Oxygen saturation (%)	98 (96 - 99)
White blood cells, 10^3/uL	8.3 (6.09 - 11.3)
Hemoglobin, g/dL	12.8 (11.2 - 14.2)
Platelets, 10^3/uL	220 (169 - 281)
Sodium, mmol/L	138 (135 - 140)
Potassium, mmol/L	4.2 (3.8 - 4.6)
Chloride, mmol/L	103 (100 - 106)
Blood urea nitrogen, mg/dL	16 (11 - 28)
Creatinine, mg/dL	0.95 (0.73 - 1.39)
Aspartate aminotransferase, U/L	31 (20 - 52)
Alanine aminotransferase, U/L	24 (15 - 41)
Total bilirubin, mg/dL	0.6 (0.4 - 0.9)
Albumin, g/dL	3.4 (3 - 3.9)
Calcium, mg/dL	8.8 (8.3 - 9.3)

Categorical variables are presented as counts and proportions; continuous variables are presented as medians and interquartile ranges.

Supplementary Figure 3: Feature Importance in the multi-tree XGBoost model

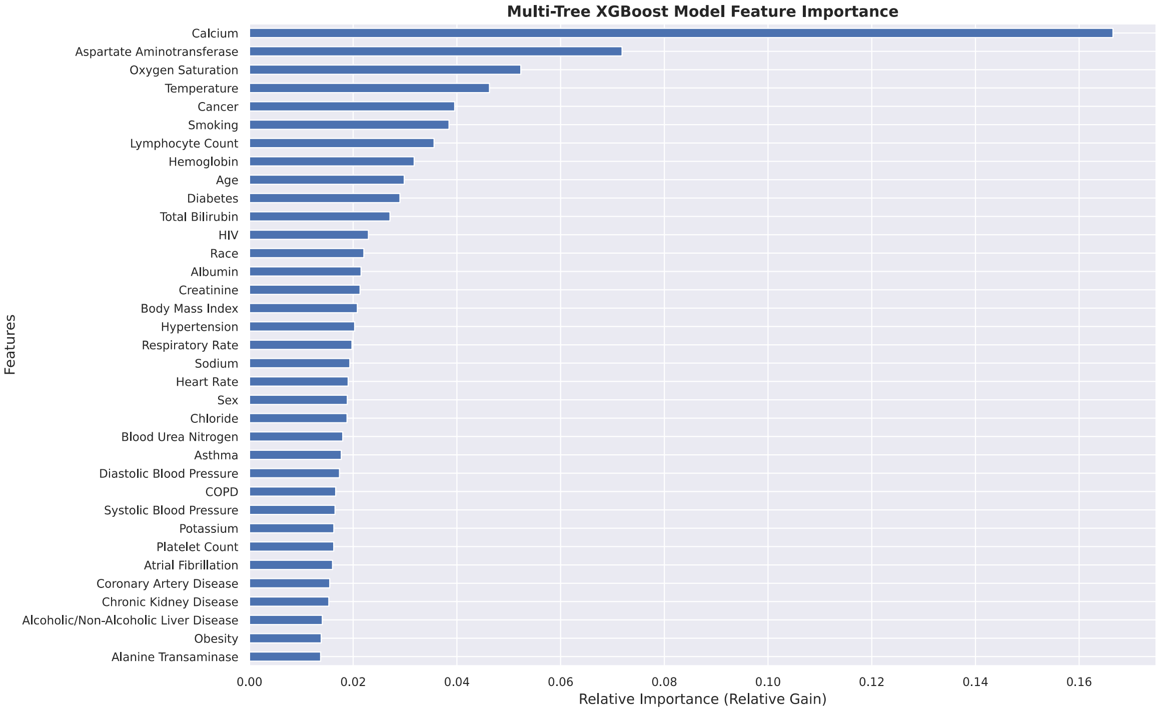

Features are ranked by relative gain (the average log loss reduction gained by using a particular feature to split the data divided by the total log loss reduction across all features).
